# Supplementary material for: Regulation of CREB Phosphorylation in Nucleus Accumbens after Relief Conditioning
Source: Cells. 2021 Jan 26;10(2):238. doi: 10.3390/cells10020238 (PMC7912172; doi:10.3390/cells10020238)
Supplement: Supplementary file 1 [file cells-10-00238-s001.pdf]

Supplementary Materials: The following are available online at www.mdpi.com/xxx/s1, Figure S1: Representative Western blot for ERK1/2, pERK1/2, CaMKIIα, and MAP2K in NAC. Figure S2: The expression level of ERK1/2, pERK1/2, CaMKIIα, and MAP2K. Figure S3: The expression level of 14-3-3 protein. Figure S4: The expression level of neuroligin2.

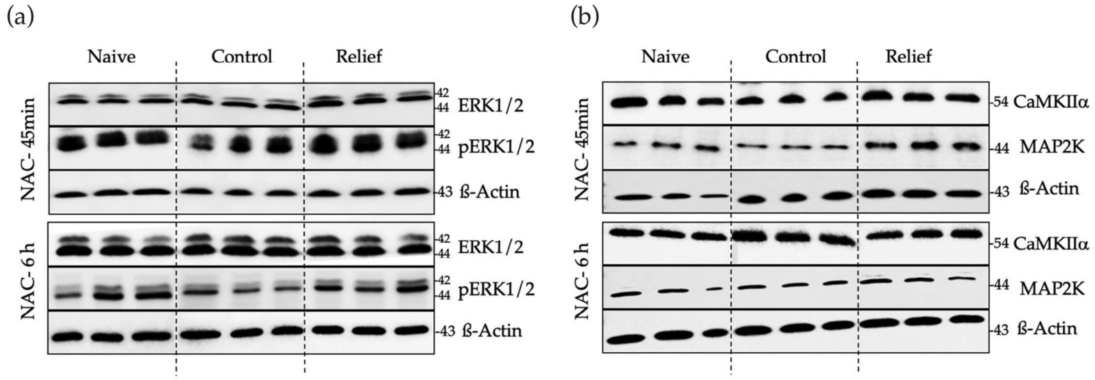

Figure S1. Representative Western blot ERK1/2, pERK1/2, CaMKIIα, and MAP2K in NAC. The expression and phosphorylation of **(a)** ERK1/2, pERK1/2, **(b)** CaMKIIα, and MAP2K in NAC showed no significant differences 45 min and 6 h after control and relief conditioning (3 different biological samples/group).

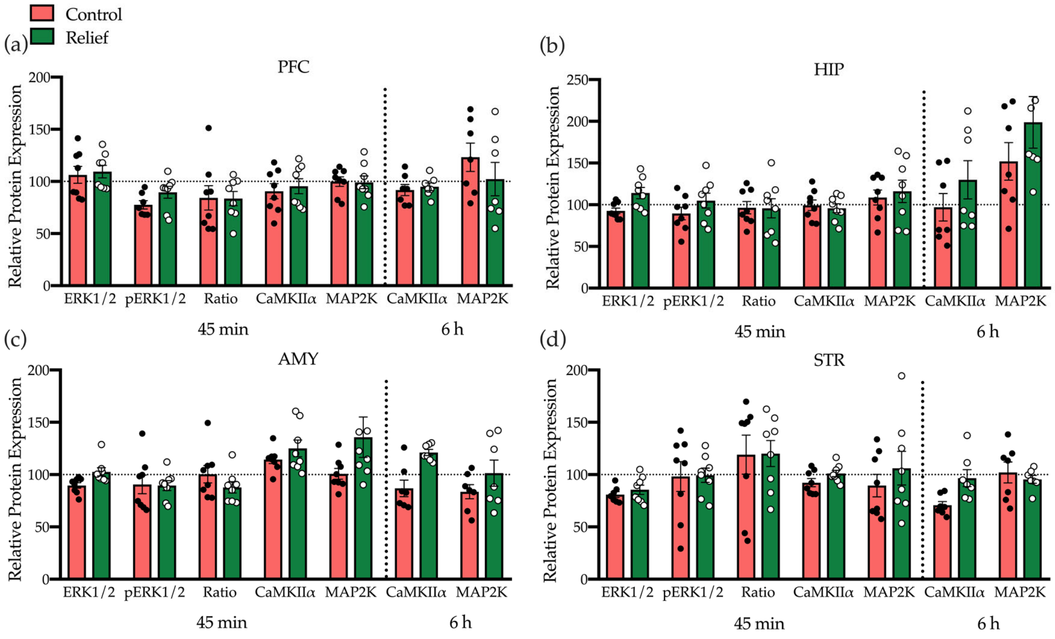

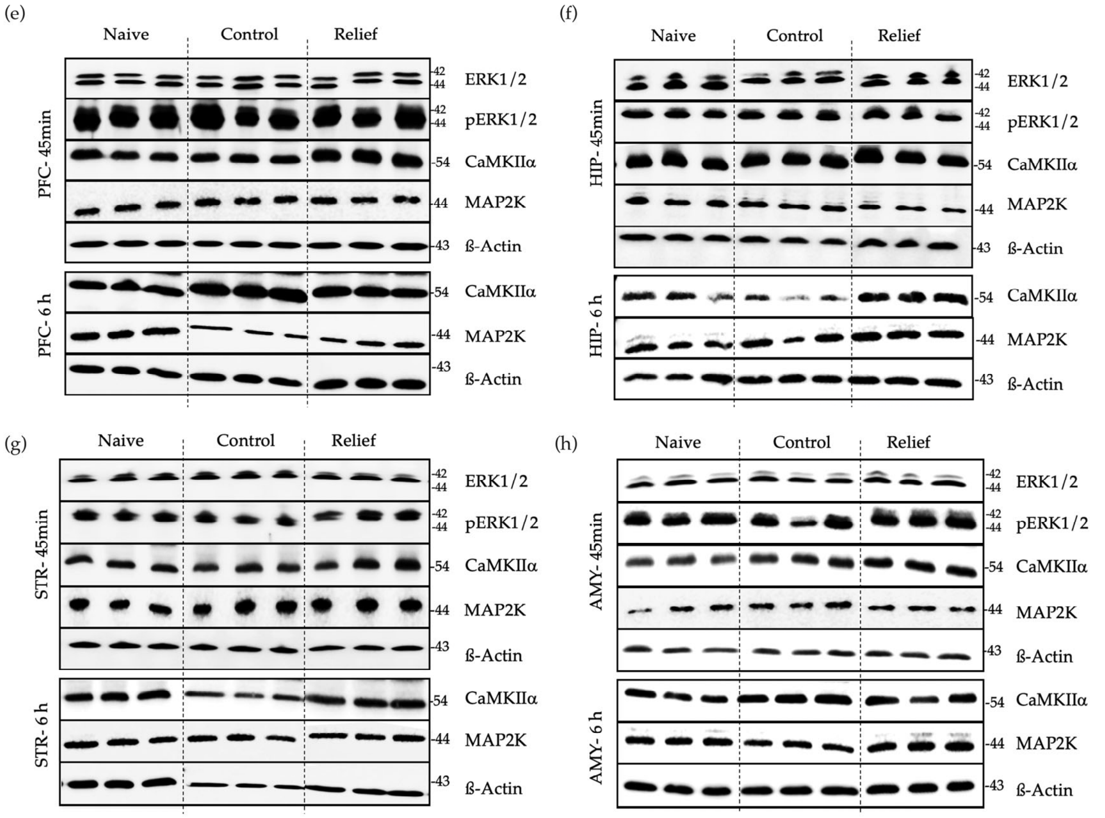

Figure S2. The expression level of ERK1/2, pERK1/2, CaMKIIα, and MAP2K. The evaluation of the selected kinases showed no significant differences between control and relief conditioned groups in **(a)** PFC, **(b)** HIP, **(c)** AMY, and **(d)** STR. Bar diagrams depict the mean ± SEM (n= 4-7). **(e-h)** Representative Western blot in PFC, HIP, AMY, and STR (3 different biological samples/group).

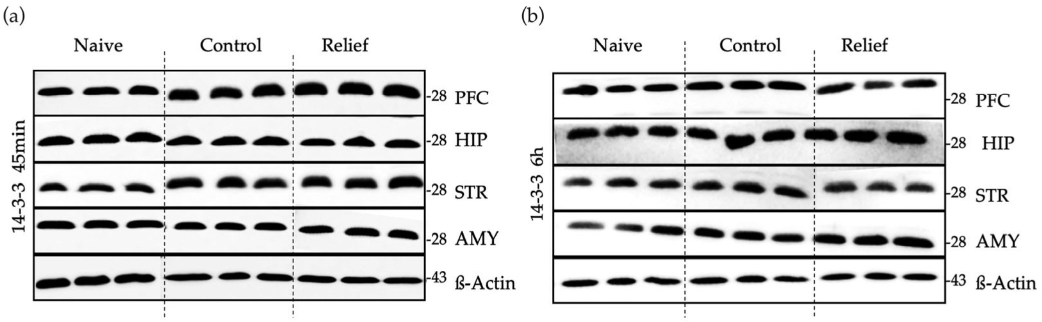

Figure S3. The expression level of 14-3-3 protein. Representative Western blot, showing the expression levels of 14-3-3, **(a)** 45 min and **(b)** 6 h following control or relief conditioning in PFC, HIP, STR, and AMY(3 different biological samples/group).

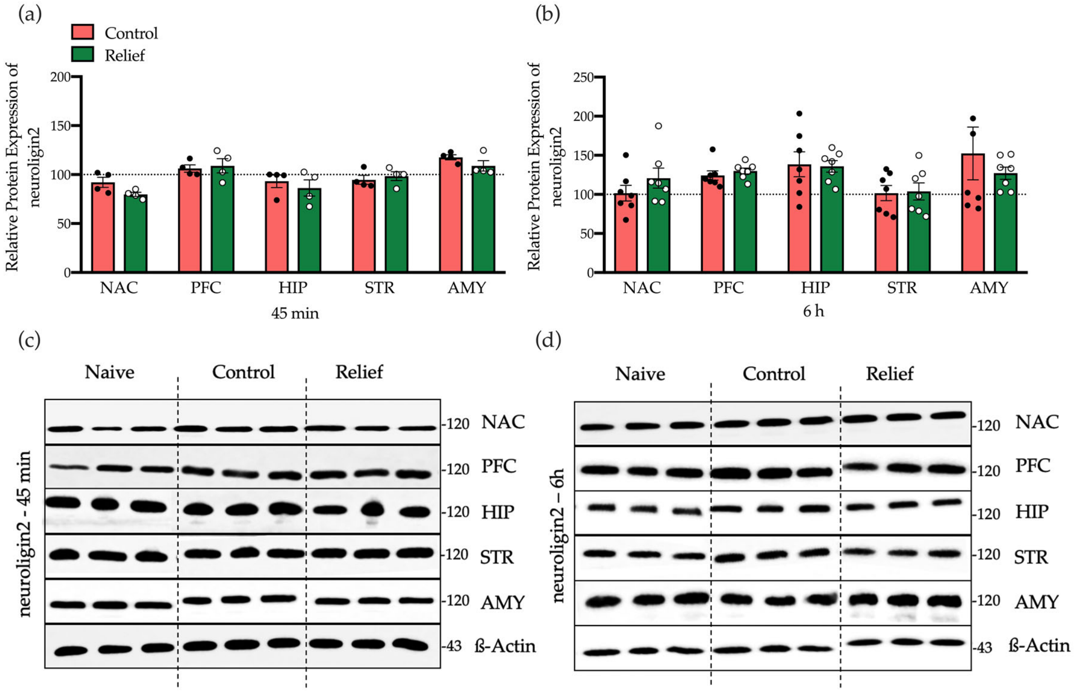

Figure S4. The expression level of neuroligin2. The expression of neuroligin2 showed no differences in any regions of the study **(a)** 45 min, and **(b)** 6 h after relief conditioning. Bar diagrams depict the mean ± SEM (*n*= 4-7/ group). **(c, d)** Representative Western blot, showing the expression levels of neuroligin2, 45 min and 6 h following control or relief conditioning in NAC (3 different biological samples/group).
